# Complete Coding Sequence of Zika Virus from a French Polynesia Outbreak in 2013

**DOI:** 10.1128/genomeA.00500-14

**Published:** 2014-06-05

**Authors:** Cécile Baronti, Géraldine Piorkowski, Rémi N. Charrel, Laetitia Boubis, Isabelle Leparc-Goffart, Xavier de Lamballerie

**Affiliations:** a Aix Marseille Université, IRD French Institute of Research for Development, EHESP French School of Public Health, EPV UMR_D 190 Emergence des Pathologies Virales, Marseille, France; b IHU Méditerranée Infection, APHM Public Hospitals of Marseille, Marseille, France; c French National Reference Centre for Arboviruses, IRBA, Marseille, France

## Abstract

Zika virus is an arthropod-borne *Flavivirus* member of the Spondweni serocomplex, transmitted by *Aedes* mosquitoes. We report here the complete coding sequence of a Zika virus strain belonging to the Asian lineage, isolated from an infected patient returning from French Polynesia, an epidemic area in 2013/2014.

## GENOME ANNOUNCEMENT

Zika virus (ZIKV) (family *Flaviviridae*, genus *Flavivirus*) is an arbovirus originally transmitted in Africa through a sylvatic cycle involving mainly *Aedes* vectors and nonhuman primates, with humans being occasional hosts (1). Epidemic transmission in a dengue-like *Aedes*-human-*Aedes* cycle has been increasingly reported in recent years (2), with nonspecific clinical presentation (influenza-, dengue- or chikungunya-like syndromes) (3).

ZIKV is phylogenetically and antigenically related to Spondweni virus, and based on nonstructural 5 (NS5) gene sequences, three lineages that reflect the East African, West African, or Asian geographic origins have been identified. The expanding distribution area of the virus makes Zika fever an emerging infectious disease, as confirmed by its spread to French Polynesia (FP) since October 2013 (>8,700 suspected and >400 laboratory-confirmed cases) (4) (http://wwwnc.cdc.gov/travel/notices/watch/zika-fever-french-polynesia-tahiti). The only previous outbreak reported in the Pacific region was on Yap island in 2007 (5).

In November 2013, a 51-year-old woman returning from FP was hospitalized in metropolitan France with fever, headache, myalgia, arthralgia, and rash. ZIKV infection was diagnosed using specific real-time reverse transcription-PCR (RT-PCR) (5). ZIKV strain H/PF/2013 isolated onto Vero cells from the serum of the patient was made available by the European Virus Archive (EVA). Viral RNA extracted from the cell culture supernatant at passage 3 was used for next-generation sequencing (Ion Torrent, Life Technologies SAS and CLC Genomics Workbench software; CLC bio) following nonspecific amplification; 137,311 reads produced a 10,617-nucleotide (nt)-long consensus contig, including the virus complete open reading frame (ORF) sequence (10,272 nt). The ORF encodes a polyprotein with three structural proteins, capsid (105 amino acids [aa]), premembrane/membrane (187 aa), and envelope (505 aa, including the envelope-154 glycosylation motif previously associated with virulence [3]), and seven nonstructural proteins, NS1 (352 aa), NS2A (217 aa), NS2B (139 aa), NS3 (619 aa), NS4A (127 aa), NS4B (255 aa), and NS5 (904 aa). The cleavage sites are identical to those reported previously. Partial 5′ and 3′ noncoding region (NCR) sequences were obtained (46/107 and 297/428 nt long, respectively, with reference to previous sequences of ZIKV isolates).

Maximum likelihood phylogenetic reconstruction (GTR + G + I model, determined from the data set using the MEGA6 program) indicated that it belonged to the Asian lineage, sharing common ancestorship and ca. 99.9% nt and aa identities with isolates circulating in southern Asia and the Pacific islands in the late 2000s (accession no. JN860885 in Cambodia, 2010, and accession no. EU545988 in Micronesia, 2007). This points to the spread of the Asian lineage, which has been suggested to originate from the introduction of ZIKV in Southeast Asia around 1945 (3, 6).

*In silico* analysis predicted efficient detection of the FP strain genome by previously published RT-PCR systems, with no mismatch for systems 835-911c (5), 9271-9373 (7), and ZIKVF9027-ZIKVR9197c (8) and one mismatch for systems ZIKVENVF-ZIKVENVR in the reverse primer (residue 16/20) (9) and 1086-1162c in probe ZIKV_1107 (residue 19/31) (5).

### Nucleotide sequence accession number.

The virus genome sequence described here has been deposited in the GenBank database under the accession no. KJ776791.
